# A numerical integrated flow-stress processing model for plain weave textile composites

**DOI:** 10.1038/s41598-025-21928-0

**Published:** 2025-10-30

**Authors:** Weijia Chen, Bin Zhang

**Affiliations:** Department of Mechanical Engineering, Suzhou University of Technology, Changshu, 215500 Jiangsu Province China

**Keywords:** Processing model, Micromechanics, Residual stress, Engineering, Materials science

## Abstract

This work proposes a numerical integrated flow-stress processing model in order to simulate the flow front of the resin and manufacturing-induced deformation during the fabrication cycle. In the flow model, each layer of the dry fiber fabric is modeled as an orthotropic porous material. The resin flow behavior is predicted using the volume of fluid method, incorporating the temperature- and cure-dependent viscosity of the resin. After the infusion model, the resin filling factor and the degree of cure are transferred to a multi-physics curing model to predict the residual stress. In this model, each lamina is modeled as a homogeneous orthotropic material with mechanical properties calculated from the in-situ resin modulus and the elastic modulus of the fiber based on the micromechanics. The composite properties, along with the thermal strains and chemical shrinkage, are incorporated into an orthotropic constitutive law to predict the residual stress. The accuracy of the proposed model is validated through comparison between the spring-in angle predicted by the numerical model and the experimental results.

**Supplementary Information:**

The online version contains supplementary material available at 10.1038/s41598-025-21928-0.

## Introduction

 Fiber-reinforced composites have been widely employed in the aerostructures because of the high strength-to-weight ratio and design flexibility. However, manufacturing of these composite structures starting from the liquid resin involves two steps: (1) the fibrous preform is thoroughly saturated by the resin fluid; (2) the resin matrix undergoes transformation from a viscous fluid into a fully-cured solid. The improper handling of infusion can generate dry spots^1,2^ and voids^3,4^ on composite structures, and the mechanical performance can be compromised. After the infusion, a cure cycle is commonly applied on composite structures to reduce the curing time of the resin. Due to the mismatch of thermo-mechanical properties of the fiber and resin, residual stress will develop inside the composite structure in the curing process, causing the warpage of non-symmetric panels^5,6^ and spring-in of curved composite structures^7,8^. At the microscopic scale, the residual stress can induce the stress concentration, which can initiate the matrix cracking^9,10^, thereby compromising the mechanical performance of composite structures. Therefore, it is essential to develop a physics-based integrated processing model to predict the potential defects and residual stress development in the composite fabrication cycle.

The resin infusion process involves a multiphase flow problem. The most straightforward method is to solve the Navier-Stokes equations separately in the resin and air phase, and the flow front of the resin can be identified according to the filling factor (volume fraction) of the resin phase in each element^11,12^. In Vacuum Assisted Resin Transfer Molding (VARTM) process, pore pressure of the resin fluid and the compaction stress sustained by the fibrous preform equilibrate the atmospheric force after the air is evacuated inside the vacuum bag. When the resin fluid enters the fibrous preform, the pore pressure rises, further reducing the compaction imposed on the fibrous preform. As a consequence, the fibrous preform is relaxed, which changes the porosity and permeability of the fibrous structure and influences the flow behavior of the resin^13–15^. Therefore, the flow front of the resin can be accurately predicted only by taking account for the coupling between the resin’s motion and compaction of the fibrous preform. On the experimental side, the most direct method for measuring the flow front in the infusion experiment is through visual inspection^11,16^, but a significant variation of flow front progression can be observed through the thickness when the fibrous preform is thick^17^. Since the mold is usually opaque, the pressure sensors can be used to monitor the flow front at the tool-part interface^18^.

In the curing process, the thermal strain and chemical shrinkage are considered as two major factors that contribute to the residual stress^19^. Considering the dependence of thermal and chemical strains on both the temperature and Degree of Cure (DOC) inside the composite, the residual stress is commonly predicted using a multi-physics process modeling framework^5^, in which the thermal analysis is performed considering the resin cure kinetics^20^ prior to the stress analysis. Based on the results provided by the thermal analysis, thermal and chemical strains are incorporated into a cure-dependent constitutive law to determine the residual stress increment^5^. Currently in literature, one of the most prevailing models to predict the development of the residual stress is the “Cure-Hardening Instantaneously Linear Elastic” (CHILE) model proposed by Johnston^21^. Although the elastic CHILE model can offer dependable predictions, it tends to over-predict the cure-induced stresses since it fails to take account for the stress relaxation of the resin when the curing temperature is above the vitrification temperature^22,23^. Several thermo-viscoelastic models have been proposed focusing on the pure resin or prepregs^24–26^, but limited models address the viscoelasticity of the composite manufactured via the liquid composite molding method, in which the direct characterization of composite mechanical response remains challenging.

Micromechanics models are commonly utilized to develop macroscopic constitutive relations based on the microstructures when analyzing fiber-reinforced composites^27^. The fiber-reinforced composite can be modeled as a homogeneous orthotropic material with its mechanical properties derived from properties of constituents using micromechanical models such as Concentric Cylinder Assemblage (CCA)^28^, the Generalized Self-Consistent Method (GSCM)^29^, and the Mori-Tanaka(M-T) theory^30^. The macroscopic stress and deformation can be predicted accurately and efficiently by utilizing these effective properties in the stress analysis. When analyzing the failure behavior of the composite structures, the microscopic stress distributions at the subscale can be reconstructed from the macroscopic stress distributions through the micromechanics tool, and the failure of the structures can be predicted^31^.

 In this paper, a numerical flow-stress processing model is proposed to simulate the resin flow movement and cure-induced residual stress of textile composite structures in the fabrication cycle. In the infusion model, the resin flow front inside the fibrous preform is predicted using the Volume of Fluid (VOF) method, which is demonstrated in Sect. "Methods for developing the flow model". The infusion model can provide predictions on the resin filling factor and DOC at the end of the infusion process, and these critical parameters are passed into a curing model to compute the residual stress. In the curing model, the residual stress is predicted through a micromechanics-based multi-physics framework, as introduced in Sect. "Methods for developing the curing model". The proposed integrated flow-stress model is incorporated into commercial software STAR-CCM + and Abaqus to predict the flow response in the infusion stage and cure-induced spring-in angle of a textile composite flange after demolding, which is presented in Sect. "Results and discussion". The accuracy of this proposed processing model is assessed through comparison between the spring-in angle predicted by the processing model and the experimental results.

## Methods for developing the flow model

During the resin infusion into fibrous preform, the flow dynamics of the resin fluid and air obeys the governing principles of the Navier-Stokes equation^32^. The advancing flow front of resin can be computationally determined by independently solving the governing Navier-Stokes equations within each phase domain (resin and air), and imposing the continuity conditions at the fluid interfaces. However, the VOF method exhibits superior computational efficiency by solving a reduced set of governing equations when addressing the multiphase flow problem. In the VOF method, the resin-air mixture is modeled as an effective single-phase fluid with its properties dependent on the filling factor of the resin in each element (*S*_*l*_) as^33^,

1 $$\begin{aligned}\:\rho\:&={\rho\:}_{l}{S}_{l}+{\rho\:}_{a}(1-{S}_{l})\\\:\mu\:&={\mu\:}_{l}{S}_{l}+{\mu\:}_{a}(1-{S}_{l})\\\:{C}_{p}&=\frac{{C}_{p,l}{\rho\:}_{l}}{\rho\:}{S}_{l}+\frac{{C}_{p,a}{\rho\:}_{a}}{\rho\:}(1-{S}_{l})\end{aligned}$$.

where *ρ*, *µ*, and *C*_*p*_ represent density, viscosity, and specific heat, respectively. The subscript “*l*” and “*a*” denote the material properties of the resin and air. The homogenized fluid properties obtained from Eq. (1) are incorporated into the Navier-Stokes equation to predict the effective flow pressure, velocity, and temperature field of the homogenized fluid.

In addition, an interface tracking equation is employed to determine the resin filling factor and resolve the resin-air interface as^33^,

2 $$\:\frac{\partial\:}{\partial\:t}\int\:{S}_{l}dV+\oint\:{S}_{l}\varvec{u}\cdot\:d\varvec{A}=\int\:-\frac{{S}_{l}}{{\rho\:}_{l}}\frac{D{\rho\:}_{l}}{Dt}dV-\int\:\frac{1}{{\rho\:}_{l}}\nabla\:\cdot\:\left({S}_{l}{\rho\:}_{l}{\varvec{u}}_{d,l}\right)dV$$

where ***u***_*d, l*_ is the resin diffusion velocity. Based on the solved resin filling factor, the pressure, velocity and temperature field of the resin can be back-calculated from those of the resin-air mixture.

Different from other multiphase flow problems, when solving the resin flow response, the curing effect should be taken into consideration as the resin viscosity experiences a substantial increase during the cure. The progress of cure is dictated by the DOC, and it can be predicted based on previous temperature history using a cure kinetics model as^5,34^,

3 $$\:\dot{\varPhi\:}=\left[{A}_{1}\text{exp}\left(-\frac{\varDelta\:{E}_{1}}{RT}\right)+{A}_{2}\text{e}\text{x}\text{p}(-\frac{\varDelta\:{E}_{2}}{RT}){\varPhi\:}^{m}\right]{(1-\varPhi\:)}^{n}$$

where *φ* is the DOC, *ΔE*_*1*_ and *ΔE*_*2*_ are the activation energy, *R* is the universal gas constant, *T* is the temperature, and *A*_*1*_, *A*_*2*_, *m*, and *n* are the cure kinetics parameters. The internal heat generated in the curing process can be expressed as,

4 $$\:{S}_{E}={\rho\:}_{l}{H}_{r}\frac{d\varPhi\:}{dt}$$

where *H*_*r*_ is the resin reaction of heat. Internal heat generation calculated from Eq. (4) is included in the Navier-Stokes equation to account for the influence of resin curing on the temperature field.

Meanwhile, the viscosity of the resin is a function of the DOC and temperature as^35^,

5 $$\:\mu\:={\mu\:}_{\infty\:}\text{e}\text{x}\text{p}(\frac{U}{RT}+{k}_{1}\varPhi\:+{k}_{2}{\varPhi\:}^{2})$$

where *µ*_*∞*_, *U*, *k*_*1*_, and *k*_*2*_ are constants in the viscosity model. It is evident that both temperature and the DOC can influence the flow movement through viscosity, and the viscosity model should be integrated into the flow model.

In summary, in the flow model, the DOC is updated first according to the previous temperature history using Eq. (3). Then, the internal heat generation at the current time step is computed using Eq. (4), and the viscosity of the resin is determined based on the current temperature and the DOC using Eq. (5). Both updated resin viscosity and internal heat generation are included in the VOF model to solve the temperature, flow movement and pressure of the resin fluid before the next time increment.

## Methods for developing the curing model

As the cure reaction progresses, the residual stress develops inside the composite due to mismatch of fiber and resin thermo-mechanical properties as well as the chemical shrinkage. Therefore, in order to accurately predict the residual stress increment, it is essential to perform the thermal analysis before the stress analysis, and therefore, a multi-physics modeling framework is established in this work, which is presented in Sect. 3.1. When performing the stress analysis, a multiscale process modeling framework is introduced, which is demonstrated in Sect. 3.2. Each lamina is homogenized with effective material properties determined from the material properties of constituents as well as the composite microstructures.

### Multi-physics process modeling framework

An initial thermal analysis is required to predict the temperature evolution inside the composite and cure progression of the resin during the cure. Based on the temperature and DOC provided by the thermal analysis, the residual stress is computed by including the thermal strain and chemical shrinkage into a 3D orthotropic constitutive law.

#### Thermal analysis

In the curing stage, the mold is usually heated in order to expedite the crosslinking reaction of the resin. As the heat transfer inside the composite is mainly achieved through conduction, the governing equation of the thermal analysis is^20^,

6 $$\:\rho\:{C}_{p}\frac{\partial\:T}{\partial\:t}=\sum\:_{n=1}^{3}\frac{\partial\:}{\partial\:{x}_{n}}\left({k}_{n}\frac{\partial\:T}{\partial\:{x}_{n}}\right)+{\rho\:}_{m}(1-{V}_{f}){H}_{r}\frac{d\varPhi\:}{dt}$$

where $\:\rho\:$ is the composite density, $\:{C}_{p}$ is the composite specific heat, $\:{k}_{n}$ is the composite thermal conductivity along the $\:n$ direction, $\:{\rho\:}_{m}$ is the resin density, and $\:{V}_{f}$ is the fiber volume fraction. The last term in Eq. (6) accounts for the cure-induced heat generation of the resin. As the internal heat generation is closely related to the DOC, the temperature field in composite can only be predicted by solving the cure kinetics model (Eq. (3)) and heat transfer governing equation (Eq. (6)) simultaneously.

#### Stress analysis

**Fig. 1:**
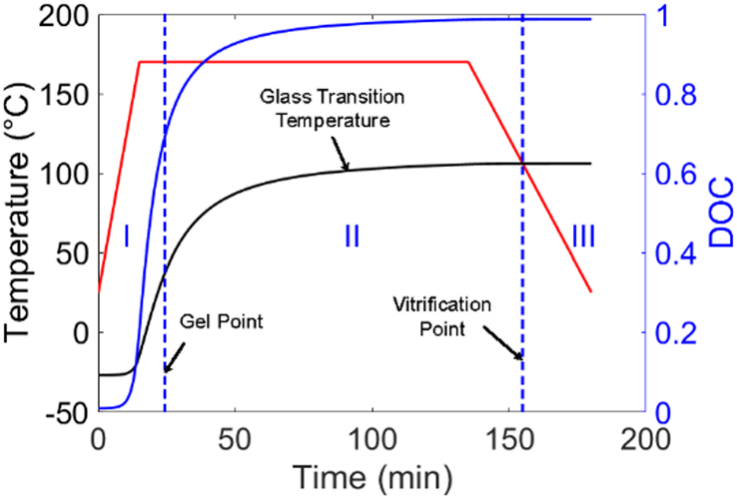
Mechanical response of a curing resin.

When a cure cycle is applied on a composite structure, the curing process of the resin starts, and the in-situ glass transition temperature grows monotonically with the DOC. The mechanical response of a curing resin can be divided into three regions, as demonstrated in Fig. 1. Before reaching the gel point, the resin manifests fluid-like behaviors, characterized by negligible load-bearing capacity. In Region Ⅱ, since the curing temperature is above the in-situ glass-transition temperature, the resin resides in a rubbery state, and the residual stress buildup can be dissipated in this stage through stress relaxation. In the cooling stage, when the curing temperature is below the glass transition temperature, the resin turns into a glassy material. In this work, a thermos-elastic model is employed to capture the residual stress increment in Region Ⅲ, which accounts for predominant portion of the total residual stress generated in the curing process. With the temperature field and DOC evolution provided by the thermal analysis, the thermal strain and chemical shrinkage are computed and incorporated into an orthotropic constitutive law to predict the residual stress as,

7 $$\:\varvec{\sigma\:}=\varvec{C}(\varvec{\epsilon\:}-{\varvec{\epsilon\:}}^{th}-{\varvec{\epsilon\:}}^{ch})$$

where $\:\varvec{\sigma\:}$ is the total stress, $\:\varvec{C}$ is the stiffness tensor of each lamina, $\:{\varvec{\epsilon\:}}^{th}$ is the thermal strain tensor, and $\:{\varvec{\epsilon\:}}^{ch}$ is the chemical strain tensor induced by the cure shrinkage. It is worthy of note that the thermal and mechanical properties in Eq. (6) and Eq. (7) are composite properties dependent on fiber and resin properties as well as the microstructures of the composite, and computation of these effective composite properties relies on a multiscale modeling framework.

### Multiscale process modeling framework

**Fig. 2:**
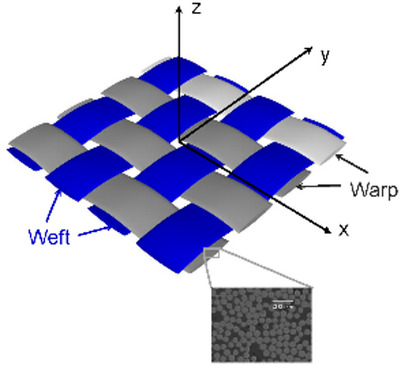
Illustration of the microstructures of a plain weave fabric.

In this work, the textile plain weave composite is modeled as a homogenous material with its thermal and mechanical properties predicted using micromechanics models and the coordinate transformation. Figure 2 demonstrates a schematic drawing depicting the microstructure of a plain weave textile fabric. The in-plane “*x*” and “*y*” axis correspond to the weft and warp tow orientations, respectively, while the “*z*” axis denotes the through-thickness direction. For the purposes of this study, fiber tow cross-sections are modeled as elliptical shapes, and the tow undulating paths are mathematically approximated using sinusoidal functions.

**Fig. 3:**
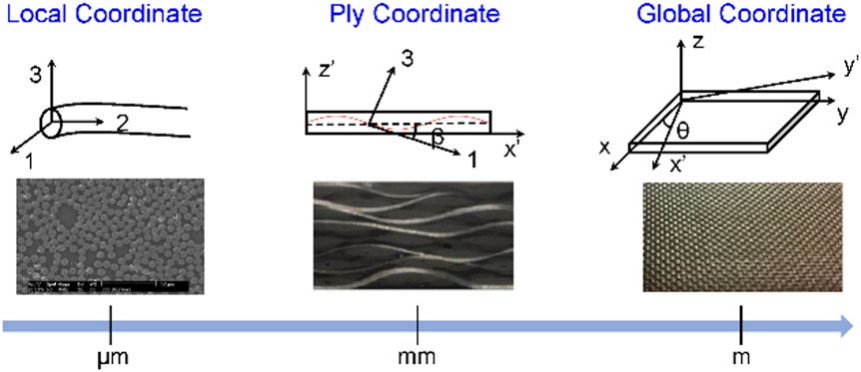
Different coordinate systems defined in plain weave textile composites.

Considering the microstructures of the plain weave composite, three coordinates systems are introduced to compute the effective material properties at different scale levels, as demonstrated in Fig. 3. A single weft or warp tow is made of unidirectionally-aligned carbon fibers surrounded by the matrix. A local coordinate is introduced with its “1” direction aligned with the fiber direction, and “2” and “3” are transverse directions, and in this coordinate, the fiber tows are modeled as unidirectionally-aligned composites. A ply coordinate is introduced at the fiber tow scale, in which “z’” axis is aligned with the ply-thickness direction of the lamina, and the fiber tow is in the “x’-z’” plane. In addition, a global coordinate, which is designated as the “x-y-z” frame, is employed as demonstrated in Fig. 2.

#### Thermal properties of the composite

It is worthy of mentioning that bulk properties of the composite, such as density and specific heat, do not need to be calculated through the coordinate transformation. Instead, these composite properties are only dependent on the properties of constituents and the fiber volume fraction as^5^,

8 $$\begin{aligned} \:{\rho\:}_{c}&={\rho\:}_{f}\left({V}_{0^\circ\:}{V}_{f,0^\circ\:}+{V}_{90^\circ\:}{V}_{f,90^\circ\:}\right)+{\rho\:}_{m}(1-{V}_{0^\circ\:}{V}_{f,0^\circ\:}-{V}_{90^\circ\:}{V}_{f,90^\circ\:})\\\:{C}_{p,c}&=\frac{{\rho\:}_{f}{C}_{p,f}\left({V}_{0^\circ\:}{V}_{f,0^\circ\:}+{V}_{90^\circ\:}{V}_{f,90^\circ\:}\right)+{\rho\:}_{m}{C}_{p,m}(1-{V}_{0^\circ\:}{V}_{f,0^\circ\:}-{V}_{90^\circ\:}{V}_{f,90^\circ\:})}{{\rho\:}_{f}\left({V}_{0^\circ\:}{V}_{f,0^\circ\:}+{V}_{90^\circ\:}{V}_{f,90^\circ\:}\right)+{\rho\:}_{m}(1-{V}_{0^\circ\:}{V}_{f,0^\circ\:}-{V}_{90^\circ\:}{V}_{f,90^\circ\:})}\end{aligned}$$.

where *ρ*_*c*_, *ρ*_*f*_, and *ρ*_*m*_ are the densities of the composite, fiber and resin, and *C*_*p, c*_, *C*_*p, f*_, and *C*_*p, m*_ are the specific heat of the composite, fiber and resin. *V*_*0°*_ and *V*_*90°*_ represent the volume fractions of the weft and warp tows, and *V*_*f,0°*_ and *V*_*f,90°*_ denote fiber volume fractions of these fiber tows.

As for thermal conductivities, in the local coordinate, the weft and warp tows can be treated as unidirectional composites, and their thermal conductivity tensors (***k***_*l, t*_) can be expressed using the thermal conductivities of unidirectional composites along the axial (*k*_*1,t*_) and transverse directions (*k*_*2,t*_). The explicit expressions of *k*_*1,t*_ and *k*_*2,t*_, which are derived through an Extended Concentric Cylinder Assemblage (ECCA) micromechanics model, can be found in Appendix A^5^.

**Fig. 4:**
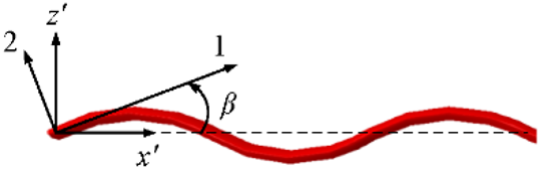
The undulating fiber tow path in x’-z’ plane.

In textile composites, assuming that the tangential vector at any given location along the fiber tow forms an angle *β* with respect to the *x’*-axis^36^, as shown in Fig. 4, and the tow undulating path can be mathematically represented by a sinusoid function, then the thermal conductivity of a fiber tow in the ply coordinate can be derived by integrating the transformed local thermal conductivity tensor over a period divided by the period length *L* as,

9 $$\:{\varvec{k}}_{p,t}=\frac{1}{L}{\int\:}_{o}^{L}{{\widehat{\varvec{T}}}_{1}}^{-1}{\varvec{k}}_{l,t}{\widehat{\varvec{T}}}_{1}dx{\prime\:}$$

where $\:{\widehat{\varvec{T}}}_{1}$ is the transformation tensor, and its analytical expression can be found in Appendix B.

The ply thermal conductivity tensors of fiber tows are then transformed into the global coordinate through a rotation about the *z/z’* axis with a fiber tow orientation angle *θ* (0° for weft tows in plain weave composites) as,

10 $$\:{\varvec{k}}_{t}={{\widehat{\varvec{T}}}_{2}}^{-1}{\varvec{k}}_{p,t}{\widehat{\varvec{T}}}_{2}$$

where $\:{\widehat{\varvec{T}}}_{2}$ is the transformation tensor with an explicit expression demonstrated in Appendix B.

Finally, the composite thermal conductivity tensor ***k*** can be determined based on the volume fractions of fiber tows and the neat resin as,

11 $$\:\varvec{k}={\varvec{k}}_{0^\circ\:}{V}_{0^\circ\:}+{\varvec{k}}_{90^\circ\:}{V}_{90^\circ\:}+{\varvec{k}}_{m}(1-{V}_{0^\circ\:}-{V}_{90^\circ\:})$$

where ***k***_*0°*_ and ***k***_*90°*_ are the thermal conductivity tensors of the weft and warp tows, and ***k***_*m*_ is the thermal conductivity tensor of the neat resin in the global coordinate.

#### Effective stiffness tensors of the composite

The stiffness of the textile composite can be determined using a similar approach. In the local coordinate, the fiber tows are modeled as unidirectionally-aligned fiber-reinforced composites. Five independent mechanical constants (*E*_*1,t*_, *E*_*2,t*_, *v*_*12,t*_, *G*_*23,t*_, *K*_*23,t*_) derived from the ECCA micromechanics model^31^ are employed to form the stiffness tensor of the weft or warp tows in the local coordinate (***C***_*l, t*_). The analytical expressions of these five constants can be found in Appendix A.

The stiffness tensors of fiber tows are transformed from the local coordinate into the ply coordinate through,

12 $$\:{\varvec{C}}_{p,t}=\frac{1}{L}{\int\:}_{o}^{L}{\widehat{\varvec{T}}}_{1,m}^{-1}{\varvec{C}}_{l,t}\varvec{R}{\widehat{\varvec{T}}}_{1,m}{\varvec{R}}^{-1}dx{\prime\:}$$

where $\:{\widehat{\varvec{T}}}_{1,m}$ is the transformation matrix related to the undulation angle *β*, and ***R*** is the rotation tensor considering the difference between the engineering and torsional strains. The analytical forms of $\:{\widehat{\varvec{T}}}_{1,m}$ and ***R*** tensors are presented in Appendix B.

The stiffness tensors of fiber tows are finally transformed into the global coordinate to compute the effective stiffness tensor of the composite as,

13 $$\:{\varvec{C}}_{t}={\widehat{\varvec{T}}}_{2,m}^{-1}{\varvec{C}}_{p,t}\varvec{R}{\widehat{\varvec{T}}}_{2,m}{\varvec{R}}^{-1}$$

where $\:{\widehat{\varvec{T}}}_{2,m}$ is the transformation tensor, and its correlation with fiber tow orientation *θ* is presented in Appendix B.

Given the transformed stiffness tensors of the fiber tows and resin stiffness tensor, the composite stiffness tensor is calculated using the rule of mixtures based on constituent volume fractions as,

14 $$\:\varvec{C}={\varvec{C}}_{0^\circ\:}{V}_{0^\circ\:}+{\varvec{C}}_{90^\circ\:}{V}_{90^\circ\:}+{\varvec{C}}_{m}(1-{V}_{0^\circ\:}-{V}_{90^\circ\:})$$

where ***C***_*0°*_ and ***C***_*90°*_ are the stiffness tensor of weft and warp tows in the global coordinate, and ***C***_*m*_ is the resin stiffness tensor.

#### Effective coefficients of thermal expansion (CTEs) and chemical strains of the composite

Computing the effective CTEs of the plain weave textile composite is challenging, since the effective CTEs are not only dependent on the CTEs of each constituent, but also dependent on their mechanical properties. In this work, the plain weave composite are approximated as a laminate with a symmetric layup of [0°/90°]_s_, and the composite CTEs are derived using Classical Laminated Plate Theory (CLPT)^5^.

The effective CTEs of a unidirectionally-aligned lamina can be determined through the ECCA model, as shown in Appendix A^5^. Based on CLPT, the constitutive relation of the laminate with symmetric layup under the thermal loading can be expressed as^37^

15 $$\:\left\{{\epsilon\:}^{0T}\right\}={\left[A\right]}^{-1}\left\{{N}^{T}\right\}$$

where *{ε*^*0T*^*}* is the mid-plane strain, *[A]* is the reduced stiffness tensor defined in CLPT, and *{N*^*T*^*}* is the thermal loading. The CTEs of the plain weave composites can be calculated using the ratio of thermal strain and temperature increment as,

16 $$\:\left\{\alpha\:\right\}=\frac{1}{\varDelta\:T}\left\{{\epsilon\:}^{0T}\right\}={\left[A\right]}^{-1}\sum\:_{k=1}^{N}{\left[\stackrel{-}{Q}\right]}_{k}{\left\{\alpha\:\right\}}_{k}{t}_{k}$$

where *ΔT* is the temperature increment, $\:{\left[\stackrel{-}{Q}\right]}_{k}$ is the reduced stiffness tenor of the *k*^*th*^ lamina in CLPT, *{α}*_*k*_ is the CTE tensor of the *k*^*th*^ lamina defined in the global coordinate, and *t*_*k*_ is the thickness of *k*^*th*^ lamina. It is worthy of mentioning that the CTEs calculated from Eq. (16) are the in-plane CTEs of textile composites. When predicting the composite through-thickness CTE, noted that the through-thickness CTEs of each lamina are identical, it is reasonable to assume that the through-thickness CTE of the composite should be equal to that of a lamina.

The chemical strains of the textile composites can be computed using a similar approach. The axial and transverse chemical strains of a fiber tow in the local coordinate can be derived using the ECCA model as^5^,

17 $$\:\varDelta\:{\epsilon\:}_{1,l}^{ch}=\frac{{\lambda\:}_{13}}{\varDelta\:}\left[{\left(1+{v}_{sh}^{T}\varDelta\:\varPhi\:\right)}^{\frac{1}{3}}-1\right](1-{V}_{f})$$

$$\:\varDelta\:{\epsilon\:}_{2,l}^{ch}=\frac{{\lambda\:}_{23}}{\varDelta\:}\left[{\left(1+{v}_{sh}^{T}\varDelta\:\varPhi\:\right)}^{\frac{1}{3}}-1\right](1-{V}_{f})$$.

where *Δε*_*1,l*_^*ch*^ and *Δε*_*2,l*_^*ch*^ represent the chemical strains of fiber tows along the axial and transverse direction, *Δφ* is the DOC increment, and *v*_*sh*_^*T*^ is the total volumetric shrinkage of the neat curing resin. Applying the CLPT, the chemical strains of the composite can be calculated by,

18 $$\:\left\{\varDelta\:{\epsilon\:}^{ch}\right\}={\left[A\right]}^{-1}\sum\:_{k=1}^{N}{\left[\stackrel{-}{Q}\right]}_{k}{\left\{\varDelta\:{\epsilon\:}^{ch}\right\}}_{k}{t}_{k}$$

where *{Δε*^*ch*^*}*_*k*_ is the chemical strains of k^th^ lamina transformed into the global coordinate. For the same reason, the through-thickness chemical strain of plain weave textile composites equals to *Δε*_*2,l*_^*ch*^ in Eq. (17).

## Results and discussion

The proposed numerical flow-stress model is utilized to predict the spring-in angle of a textile composite flange after the demolding. The accuracy of the model is validated through comparison between the predicted spring-in angle and that of a counterpart fabricated via the resin transfer molding (RTM) technique.

### Manufacturing of a composite flange

**Fig. 5:**
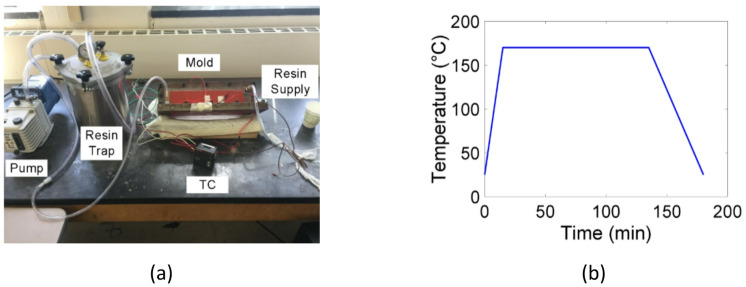
Manufacturing of the composite flange. (**a**) Experimental setup of the RTM technique, (**b**) Cure cycle used in the experiments.

A plain weave textile composite flange was manufactured using 4 K AS4 carbon fiber plain weave fabrics and EPON 862/W resin system via the RTM technique, as illustrated in Fig. 5(a). During the manufacturing procedure, 16 plies of the dry plain weave fiber fabrics were sequentially-stacked inside the mold cavity. The infusion gate on the mold was connected to resin supply, while the vent port was connected to a resin trap and a vacuum pump. The resin and mold were preheated to 60℃ using an oven and heat sheets with an aim to reduce the resin viscosity. Once the pump was started, the resultant pressure difference facilitated the viscous resin fluid infusion into the mold cavity. After the infusion was completed, a cure cycle was applied on the composite flange through two heat sheets attached to the mold, as shown in Fig. 5(b), and the temperature of the heat sheets was strictly monitored by a temperature controller. After curing was completed, the composite flange was demolded, and the spring-in angles were measured at different locations using an angle gauge. The average spring-in angle is 1.86° (± 0.18°).

### Material properties of constituents

**Table 1:** Themo-mechanical properties of the AS4 carbon fiber^5^.

Symbol	Definition	Value	Unit
*ρ*_*f*_	Density	1790	kg/m^3^
*C*_*p, f*_	Specific heat	1134	J/(kg•K)
*k*_*1,f*_	Axial thermal conductivity	6.83	W/(m•K)
*k*_*2,f*_	Transverse thermal conductivity	2.18	W/(m•K)
*E*_*1,f*_	Axial modulus	231	GPa
*E*_*2,f*_	Transverse modulus	15	GPa
*v*_*12,f*_	Axial Poisson’s ratio	0.27	-
*G*_*12,f*_	Axial shear modulus	24	GPa
*G*_*23,f*_	Transverse shear modulus	5.01	GPa
*α*_*1,f*_	Axial CTE	−9.0 × 10^−7^	K^−1^
*α*_*2,f*_	Transverse CTE	7.2 × 10^−5^	K^−1^

 In this work, the AS4 carbon fiber is treated as a transversely-isotropic material with its thermo-mechanical properties presented in Table 1^5^. Based on the literature, the fibrous preform is modeled as a transversely-isotropic porous material with in-plane permeability designated as 5.0 × 10^−10^m^2^, while the through-thickness permeability is 5.0 × 10^−11^m^[2 38^.

**Table 2:** Parameters used in the cure kinetics model of EPON 862/W resin system^39^.

Cure kinetics parameters	Characterized value
*A*_*1*_ (s^−1^)	0
*ΔE*_*1*_ (J)	-
*A*_*2*_ (s^−1^)	7098
*ΔE*_*2*_ (J)	5.50 × 10^4^
*m*	0.40
*n*	1.65
*H*_*r*_ (J/g)	406.12

**Table 3:** Thermo-mechanical properties of EPON 862/W resin system^39^.

Symbol	Definition	Value	Unit
*ρ*_*m*_	Density	1300	Kg/m^3^
*C*_*p, m*_	Specific heat	1219	J/(kg•K)
*k*_*m*_	Thermal conductivity	0.148	W/(m•k)
*α*_*m*_	CTE	7.78 × 10^−5^	K^−1^
*v*_*sh*_^*T*^	Chemical shrinkage	−3.72%	-

 For EPON 862/W resin system, the cure kinetics model was characterized using the Differential Scanning Calorimeter (DSC) technique, and the coefficients of the cure kinetics model together with its thermal properties can be found in Tables 2 and 3^39^. The in-situ cure-dependent glass transition temperature is predicted through the Dibenedetto equation as^40^,

19 $$\:\frac{{T}_{g}\left(\varPhi\:\right)-{T}_{g}^{0}}{{T}_{g}^{\infty\:}-{T}_{g}^{0}}=\frac{\lambda\:\varPhi\:}{1-\left(1-\lambda\:\right)\varPhi\:}$$

The parameters used in Eq. (19) can be found in Table 4.

**Table 4:** Parameters used in the DiBenedetto model for EPON 862/W resin system^40^.

Symbol	Value	Unit
*T*_*g*_^*0*^	−27.1	℃
*T*_*g*_^*∞*^	110	℃
*λ*	0.392	-

**Fig. 6:**
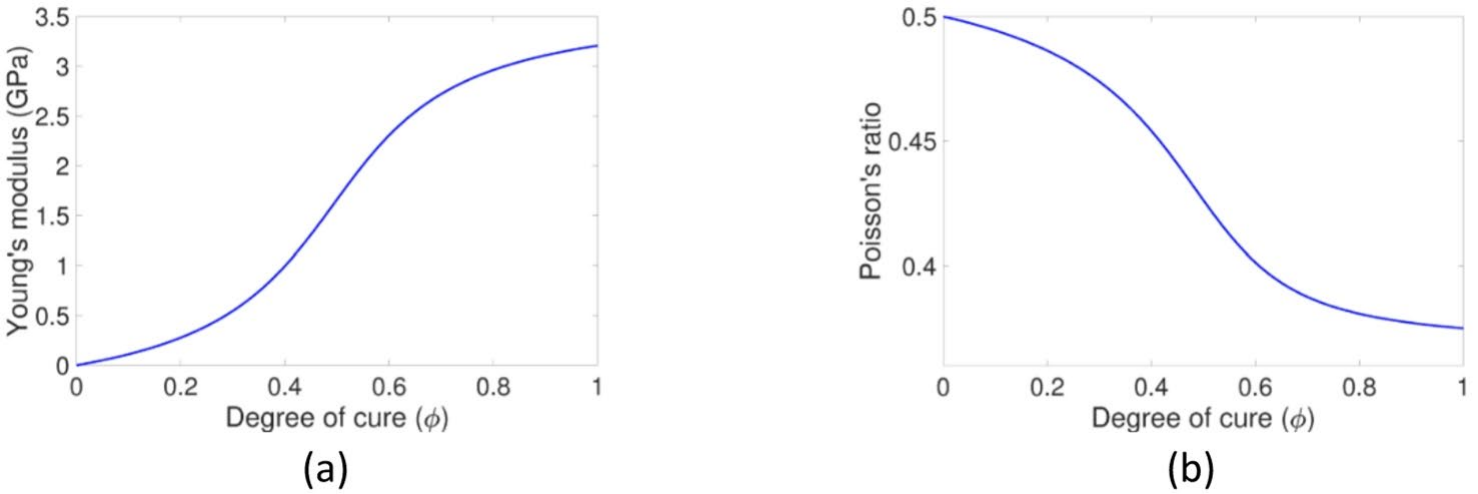
Evolution of resin mechanical properties in the curing process. (**a**) The cure-dependent Young’s modulus, (**b**) The cure-dependent Poisson’s ratio.

As for the mechanical properties, the curing resin is modeled as an isotropic material with cure-dependent mechanical properties. Therefore, only two independent mechanical properties are required to define the stiffness tensor, and in-plane bulk modulus and shear modulus are chosen since they can be directly measured using the Brillouin Light Scattering (BLS) method^21^. These two properties are then converted to the Young’s modulus and Poisson’s ratio, and their correlations with the DOC are illustrated in Fig. 6. The viscosity model employed in this work was characterized by Shanku et al. using a rheometer, and the coefficients of the viscosity model can be found in Table 5.

**Table 5:** Material constants used in the viscosity model of the resin^35^.

Symbol	Value	Unit
*µ*_*∞*_	7.70 × 10^−5^	Pa•s
*U*	2.52 × 10^4^	J
*k*_*1*_	4.47	-
*k*_*2*_	13.58	-

### Prediction of the resin flow movement and Spring-in angle using the proposed processing model

#### Prediction of the resin flow response

**Fig. 7:**
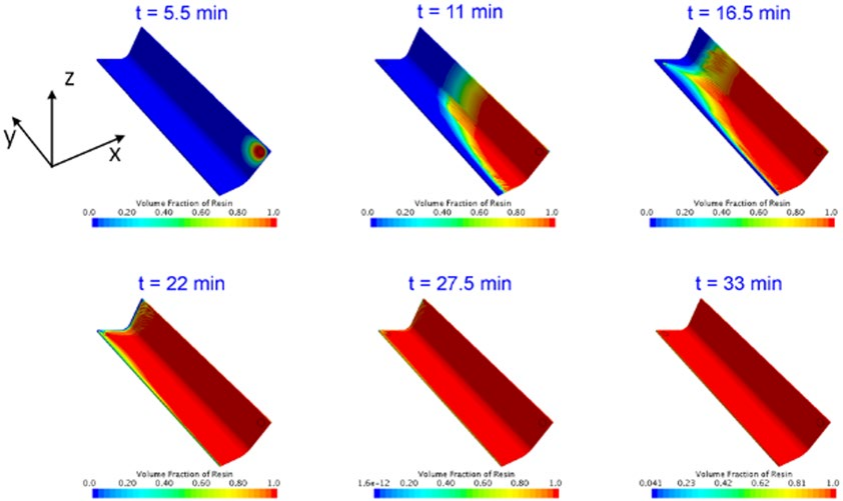
Predicted resin flow front advancement in STAR-CCM+.

A full-scale homogenized composite flange model was constructed in STAR-CCM + to predict the resin flow front advancement inside the composite. The wing length of the L-shaped flange was 76.2 mm, while the longitudinal span length was 355.6 mm. The thickness of the flange was 3.34 mm. The flange was defined as a porous domain with a porosity of 0.41, which was computed based on the fiber volume fraction. On the upper surface of the flange, two circular domains were created utilizing the “Slicing” command, and they correspond to the infusion gate and vent locations. At the infusion gate, one atmospheric pressure was imposed, and the resin volume fraction maintained at unity. At the vent, the pressure was set to be zero. Wall boundary conditions were imposed on all remaining external surfaces. A uniform temperature field of 60℃ was applied on the mold, and the inlet temperature was set to be 60℃ as well. A state variable was created to compute the DOC increment based on the temperature history using the cure kinetics model, and the viscosity of the resin was set to be a function of DOC and temperature using Eq. (5). Internal heat generation calculated using Eq. (4) was implemented into the VOF model using a C + + script. Upon completion of the flow simulation, the resin filling factor and the DOC were passed into the curing model using a C + + script. The predicted flow movement is demonstrated in Fig. 7. The result indicates that the fibrous preform is thoroughly impregnated after 33 min, and there should be no dry spots inside the composite flange, which is consistent with the experimental results.

#### Prediction of the spring-in angle through the curing model

**Fig. 8:**
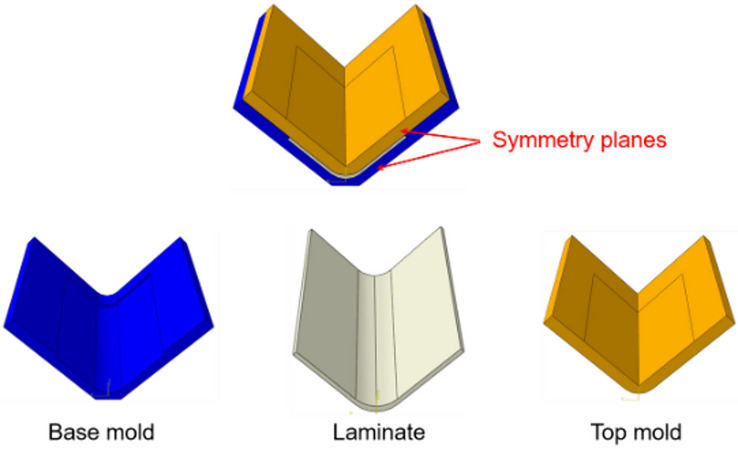
The curing finite element model created in Abaqus.

The curing model was numerically implemented into the finite element software Abaqus to simulate the residual stress in the curing process. The created finite element model is depicted in Fig. 8, and in order to demonstrate the laminate inside the cavity of the closed mold, only half of the model is displayed. The dimensions of the created model strictly follow those of the experimental setup. As the composite flange was an anisotropic material, a material coordinate “$\:x-y-z$” was created, in which the $\:y$ axis was aligned with the span length direction, and $\:z$ axis was aligned with the through-thickness direction of the composite flange. On the upper surface of the top mold and lower surface of the base mold, two heating areas were created using “Partition” command in Abaqus, and the sizes and locations of the heating areas were determined based on those of the heat sheets.

**Fig. 9:**
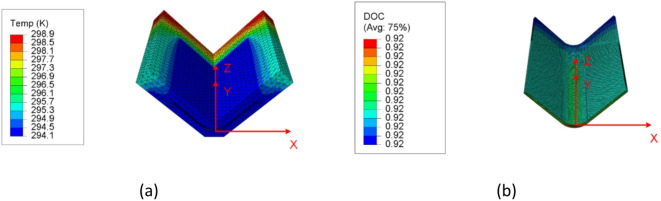
Temperature and DOC fields at the end of the cure cycle. (**a**) Temperature distribution, (**b**) DOC distribution.

The first step of the curing model is to perform the thermal analysis to predict the temperature and DOC. In Abaqus, the composite flange utilized DC3D20 quadratic solid elements for discretization, while DC3D8 linear elements were used to mesh the mold. A cure cycle depicted by Fig. 5(b) was imposed on the heating areas, and convection boundary conditions were prescribed on all remaining external surfaces of the mold. The heat transfer governing equation (Eq. (6)) considering the cure-induced heat generation and cure kinetics (Eq. (3)) were implemented into Abaqus user-defined subroutine UMATHT. At each time increment, based on the temperature history, the DOC was computed. The ECCA model and the coordinate transformation were employed to update the thermal conductivity tensor of the composite based on the fiber and resin properties as well as the microstructures. The temperature field was then solved through the heat transfer governing equation considering the internal heating generation (Eq. (6)) before the next time increment. The temperature and DOC distributions were found uniform during the curing process, as shown in Fig. 9 (only half of the model was presented with an aim to demonstrate the flange inside the mold).

**Fig. 10:**
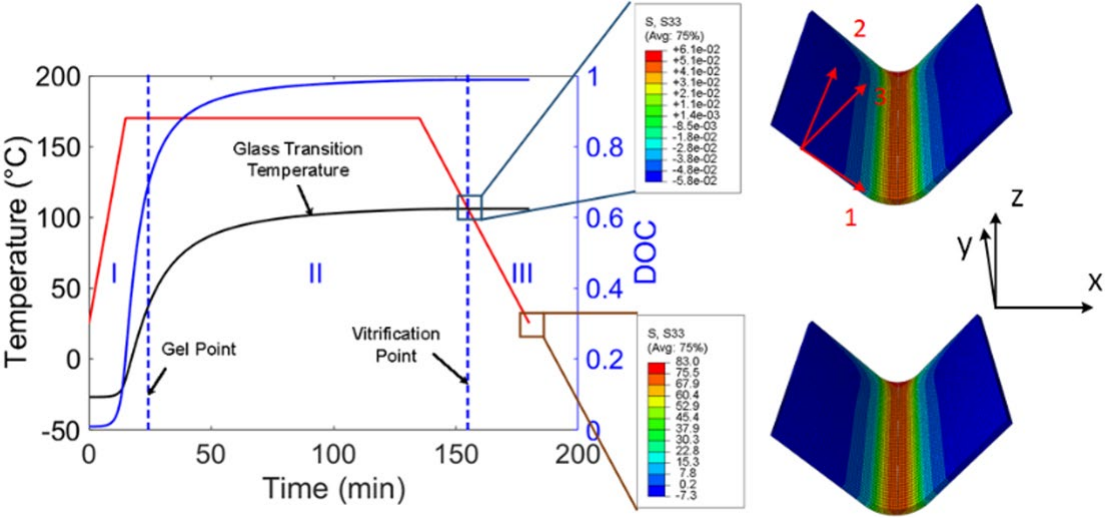
Stress distribution changes in the curing process (Unit: MPa).

The subsequent step involves predicting the residual stress, and in the stress analysis, C3D20 quadratic solid elements were employed to mesh the composite flange, and C3D8 linear solid elements were used to mesh the mold. The cure-dependent micromechanics-based constitutive law was implemented into the Abaqus user-defined subroutine UMAT. At each time increment, based on predicted temperature and DOC from the thermal analysis, the resin properties were updated. Applying the ECCA micromechanics model and the coordinate transformation, the mechanical properties, CTEs, and the chemical strains of the fiber tows and resin were obtained in the global coordinate, and the rule of mixtures were used to compute the in-situ composite effective stiffness tensor, thermal strains, and chemical strains. When the curing temperature is below the in-situ glass transition temperature of the curing resin, these effective properties were incorporated into a constitutive relation (Eq. (7)) to predict the residual stress. A “hard” tool-part interaction was defined at the composite-mold interface to avoid the geometric interference in the simulation. Stress distribution along the through-thickness direction, which was a major contributing factor to the spring-in deformation, was shown in Fig. 10 at the beginning and end of Region Ⅲ. Significant residual stress buildup can be observed at the end of the cure cycle.

**Fig. 11:**
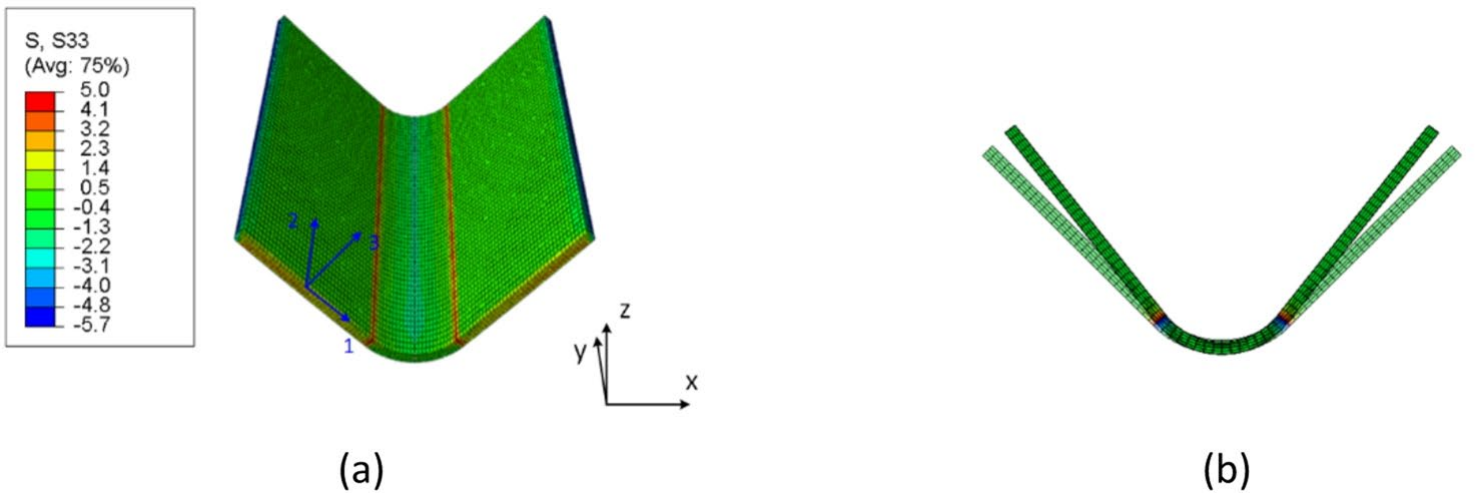
Stress distribution and structural deformation after demolding. (**a**) Stress component along the through-thickness direction after demolding, (MPa), (**b**) Spring-in occurs after demolding.

Upon completion of the curing model, a single-step demolding process was implemented to simulate the release of the flange from the tooling at the end of the fabrication cycle. The mold elements were removed via a “Model Change” command, and the tool-part interaction was deactivated as well. Once the demolding was completed, under the influence of the residual stress, the composite structure exhibited spring-in deformation, as demonstrated in Fig. 11. The predicted spring-in angle is 2.16°, which is close to but higher than the experimental results.

### Limitation and future work

The major limitation of this work is that the resin constitutive law does not account for the stress relaxation behaviors, resulting in overestimated residual stress predictions relative to the experimental results. With an aim to provide residual stress predictions with high-fidelity, it is imperative to implement the viscoelasticity of the resin into the processing model. In the time domain, as the mechanical performance of the resin matrix follows viscoelastic constitutive relations, conventional elastic micromechanics models cannot be directly applied to obtain the effective viscoelastic properties of the composites. A challenge the authors have to face is the difficulty of predicting the composite viscoelastic constitutive law from the constituent properties considering the complex microstructures of the textile composites.

## Conclusion

A numerical integrated flow-stress model was introduced to simulate the resin flow and cure-induced deformation during the fabrication cycle of the composites. The flow model employed the VOF method to solve the resin flow movement, incorporating the internal heat generation and a cure-dependent viscosity model. Based on the resin filling factor and DOC provided by the flow model, a micromechanics-based multi-physics curing model was developed to predict the residual stresses and cure-induced deformation. With provided temperature field and DOC progression via the thermal analysis, the thermal strain and chemical shrinkage were incorporated into a cure-dependent constitutive law to predict the residual stress. The accuracy of the integrated processing model was validated through comparison between the spring-in angle predicted by the numerical model and that of a counterpart manufactured using the RTM technique.

## Supplementary Information

Below is the link to the electronic supplementary material.

Supplementary Material 1

## Data Availability

The datasets used and/or analyzed during the current study are available from the corresponding author on reasonable request.

## References

[1] Shevtsov, S. et al. Two-stage numerical approach for reliable recognition of dry spots at the VAP infusion of large composite parts of complex shape. *Compos. Struct.* **259**, 113437 (2021).

[2] Chai, B. X. et al. Simulation-based optimisation for injection configuration design of liquid composite moulding processes: A review. *Compos. Part A: Appl. Sci. Manufac.* **149**, 106540 (2021).

[3] Bertling, D., Kaps, R. & Mulugeta, E. Analysis of dry-spot behavior in the pressure field of a liquid composite molding process. *CEAS Aeronaut. J.* **7** (4), 577–585 (2016).

[4] Machado, J. M. et al. Automatic void content assessment of composite laminates using a machine-learning approach. *Compos. Struct.* **288**, 115383 (2022).

[5] Chen, W. & Zhang, D. A micromechanics-based processing model for predicting residual stress in fiber-reinforced polymer matrix composites. *Compos. Struct.* **204**, 153–166 (2018).

[6] Vermes, B. & Czigany, T. Thermally induced mechanical work and warpage compensation of asymmetric laminates. *Compos. Struct.* **295**, 115847 (2022).

[7] Fiorina, M. et al. Spring-in prediction for carbon/epoxy aerospace composite structure. *Compos. Struct.* **168**, 739–745 (2017).

[8] Chen, J. et al. Analytical solutions for process-induced spring-in of U-shaped composite parts. *Thin-Walled Struct.* **169**, 108425 (2021).

[9] Corden, T. J. et al. The mechanisms of interlaminar cracking in Thick resin transfer moulded composite cylinders. *Compos. Part A: Appl. Sci. Manufac.* **29** (4), 455–464 (1998).

[10] Yuan, M. et al. Prediction of matrix-cracking-induced stiffness degradation of cross-ply laminates based on data-driven method. *Compos. Sci. Technol.* **230**, 109716 (2022).

[11] Allen, I. I. I. & Myron, B. *The Mathematics of Fluid Flow Through Porous Media* (Wiley, 2021).

[12] Vilà, J., González, C. & Javier LLorca. Fabric compaction and infiltration during vacuum-assisted resin infusion with and without distribution medium. *J. Compos. Mater.* **51** (5), 687–703 (2017).

[13] Tamakuwala, V. R. Manufacturing of fiber reinforced polymer by using VARTM process: A review. *Materials Today: Proceedings* 44 : 987–993. (2021).

[14] Govignon, Q., Bickerton, S. & Kelly, P. A. Simulation of the reinforcement compaction and resin flow during the complete resin infusion process. *Compos. Part A: Appl. Sci. Manufac.* **41** (1), 45–57 (2010).

[15] Gajjar, T. et al. Experimental study of thickness gradient and flow simulation in VARTM process. *Fibers Polym.* **21**, 384–391 (2020).

[16] Grössing, H. et al. Flow front advancement during composite processing: predictions from numerical filling simulation tools in comparison with real-world experiments. *Polym. Compos.* **37** (9), 2782–2793 (2016).

[17] Hsiao, K. T. et al. A closed form solution for flow during the vacuum assisted resin transfer molding process. *J. Manuf. Sci. Eng.* **122** (3), 463–475 (2000).

[18] Di Fratta, C. et al. Fast method to monitor the flow front and control injection parameters in resin transfer molding using pressure sensors. *J. Compos. Mater.* **50** (21), 2941–2957 (2016).

[19] Shah, S. P. et al. Process modeling and characterization of thermoset composites for residual stress prediction. *Mech. Adv. Mater. Struct.* **30** (3), 486–497 (2023).

[20] Shah, S. P., Marianna & Maiarù Effect of manufacturing on the transverse response of polymer matrix composites. *Polymers* 13.15 : 2491. (2021). 10.3390/polym13152491 PMC8348792 34372094

[21] Johnston, A. A. *An Integrated Model of the Development of process-induced Deformation in Autoclave Processing of Composite Structures* (Diss. University of British Columbia, 1997).

[22] An, N. et al. Multiscale modeling of viscoelastic behavior of unidirectional composite laminates and deployable structures. *Mater. Design*. **219**, 110754 (2022).

[23] Safarabadi, M. Evaluation of curing residual stresses in three-phase thin composite laminates considering micro-scale effects. *J. Compos. Mater.* **50**, 3753–3764 (2016).

[24] Courtois, A. et al. Viscoelastic behavior of an epoxy resin during cure below the glass transition temperature: characterization and modeling. *J. Compos. Mater.* **53** (2), 155–171 (2019).

[25] Li, H. & Zhang, B. Improved models of viscosity and relaxation modulus for epoxy resin during cure. *Polym. Eng. Sci.* **56** (6), 617–621 (2016).

[26] Ding, A. et al. A three-dimensional thermo-viscoelastic analysis of process-induced residual stress in composite laminates. *Compos. Struct.* **129**, 60–69 (2015).

[27] Aboudi, J., Arnold, S. M. & Bednarcyk, B. A. *Micromechanics of Composite Materials: a Generalized Multiscale Analysis Approach* (Butterworth-Heinemann, 2012).

[28] Hashin, Z. & Walter Rosen, B. The elastic moduli of fiber-reinforced materials. *J. Appl. Mech.* **31** (2), 223–232 (1964).

[29] Christensen, R. M. & Lo, K. H. Solutions for effective shear properties in three phase sphere and cylinder models. *J. Mech. Phys. Solids*. **27** (4), 315–330 (1979).

[30] Mori, T. & Tanaka, K. Average stress in matrix and average elastic energy of materials with misfitting inclusions. *Acta Metall.* **21** (5), 571–574 (1973).

[31] Zhang, D. & Meyer, P. Waas. An experimentally validated computational model for progressive damage analysis of Notched oxide/oxide woven ceramic matrix composites. *Compos. Struct.* **161**, 264–274 (2017).

[32] Robinson, J. C., Rodrigo, J. L. & Sadowski, W. *The three-dimensional Navier–Stokes Equations: Classical Theory*Vol. 157 (Cambridge University Press, 2016).

[33] Hirt, C. W., Billy, D. & Nichols Volume of fluid (VOF) method for the dynamics of free boundaries. *J. Comput. Phys.* **39** (1), 201–225 (1981).

[34] Heinrich, C. et al. The role of curing stresses in subsequent response, damage and failure of textile polymer composites. *J. Mech. Phys. Solids*. **61** (5), 1241–1264 (2013).

[35] Shanku, R., Vaughan, J. G. & Roux, J. A. Rheological characteristics and cure kinetics of EPON 862/W epoxy used in pultrusion. *Adv. Polym. Technology: J. Polym. Process. Inst.* **16** (4), 297–311 (1997).

[36] Zhang, D. *Progressive Damage and Failure Analysis of 3D Textile Composites Subjected to Flexural Loading*. Diss. (2014).

[37] Öchsner, A. *Foundations of Classical Laminate Theory* (Springer, 2021).

[38] Nedanov, P. B. & Suresh, G. Advani. A method to determine 3D permeability of fibrous reinforcements. *J. Compos. Mater.* **36** (2), 241–254 (2002).

[39] Chen, W. & Zhang, D. Improved prediction of residual stress induced warpage in thermoset composites using a multiscale thermo-viscoelastic processing model. *Compos. Part A: Appl. Sci. Manufac.* **126**, 105575 (2019).

[40] O’Brien, D. J., Scott, R. & White Cure kinetics, gelation, and glass transition of a bisphenol F epoxide. *Polym. Eng. Sci.* **43** (4), 863–874 (2003).

