# Supplementary material for: A numerical integrated flow-stress processing model for plain weave textile composites
Source: Sci Rep. 2025 Oct 30;15:37995. doi: 10.1038/s41598-025-21928-0 (PMC12575643; doi:10.1038/s41598-025-21928-0)
Supplement: Supplementary file 1 — Supplementary Material 1 [file 41598_2025_21928_MOESM1_ESM.docx]

**Supplementary Material**

**A Numerical Integrated Flow-Stress Processing Model for Plain Weave Textile Composites**

Weijia Chen, Bin Zhang

**Appendix A. Computation of Thermo-Mechanical Properties of Fiber Tows**

In this work, the thermal conductivities, mechanical properties, and the CTEs of fiber tows are predicted using the ECCA micromechanics model, and the derivation can be found in previous work ^[1]^. For a unidirectionally-aligned fiber tow, the thermal conductivities of fiber tows along the axial and transverse directions are expressed as ^[1]^,

| $k_{1,t}=k_{1,f}V_{f,t}+k_{m}(1-V_{f,t})$ | (A.1) |
| --- | --- |
| $k_{2,t}=\frac{\left( 1+V_{f,t} \right)k_{2,f}+(1-V_{f,t})k_{m}}{\left( 1-V_{f,t} \right)k_{2,f}+(1+V_{f,t})k_{m}}k_{m}$ |  |

where *k_1,f_* and *k_2,f_* are axial and transverse thermal conductivities of the fiber, respectively, *k_m_* is resin thermal conductivity, and *V_f,t_* is the fiber volume fraction inside the weft or warp tows.

Since the fiber tow is treated as a transversely-isotropic material, five independent mechanical properties are utilized to construct the stiffness tensor, which are ^[2]^

| $E_{1,t}=E_{1,f}V_{f,t}+E_{m}\left( 1-V_{f,t} \right)+\frac{4V_{f,t}(1-V_{f,t}){(v_{12,f}-v_{m})}^{2}G_{m}}{\frac{(1-V_{f,t})G_{m}}{K_{23,f}}+\frac{V_{f,t}G_{m}}{K_{23,m}}+1}$ | (A.2) |
| --- | --- |
| $v_{12,t}=v_{12,f}V_{f,t}+v_{m}\left( 1-V_{f,t} \right)+\frac{V_{f,t}(1-V_{f,t})(v_{12,f}-v_{m})(\frac{G_{m}}{K_{23,m}}-\frac{G_{m}}{K_{23,f}})}{\frac{(1-V_{f,t})G_{m}}{K_{23,f}}+\frac{V_{f,t}G_{m}}{K_{23,m}}+1}$ |  |
| $G_{12,t}=\frac{G_{12,f}\left( 1+V_{f,t} \right)+G_{m}(1-V_{f,t})}{G_{12,f}\left( 1-V_{f,t} \right)+G_{m}(1+V_{f,t})}G_{m}$ |  |
| $K_{23,t}=K_{23,m}+\frac{V_{f,t}}{\frac{1}{K_{23,f}-K_{23,m}}+\frac{1-V_{f,t}}{K_{23,m}+G_{m}}}$ |  |

while the fifth constant *G_23,t_* is solved from a quadratic equation ^[2]^,

| $A{(\frac{G_{23,t}}{G_{m}})}^{2}+B\left( \frac{G_{23,t}}{G_{m}} \right)+C=0$ | (A.3) |
| --- | --- |

where *A*, *B*, and *C* are functions of fiber volume fraction inside the fiber tows *V_f,t_*. Meanwhile, the axial and transverse CTEs of the fiber tow can be expressed as ^[1]^,

| $\alpha_{1,l}=\frac{1}{\Delta}\left[ \lambda_{11}\alpha_{1,f}V_{f,t}+\lambda_{12}\alpha_{2,f}V_{f,t}+\lambda_{13}\alpha_{m}({1-V}_{f,t}) \right]$ | (A.4) |
| --- | --- |
| $\alpha_{2,l}=\frac{1}{\Delta}\left[ \lambda_{21}\alpha_{1,f}V_{f,t}+\lambda_{22}\alpha_{2,f}V_{f,t}+\lambda_{23}\alpha_{m}({1-V}_{f,t}) \right]$ |  |

*Δ* and *λ* are functions of fiber and resin mechanical properties together with the fiber volume fraction of the fiber tow as,

| $\Delta=E_{1}^{f}V_{f,t}K_{23}^{f}\left( 4v_{m}V_{f,t}-2V_{f,t}-2 \right)\left( 1+v_{m} \right)+E_{1}^{f}V_{f,t}E^{m}\left( 1-V_{f,t} \right)-E^{m}(1-V_{f,t})$  $\{\left\{ \left[ \left( 8v_{12}^{f}+2 \right)v_{m}-4v_{12}^{f^{2}}-2 \right]V_{f,t}-2\left( v_{m}+1 \right) \right\}K_{23}^{f}-E^{m}\left( 1-V_{f,t} \right)\}$ | (A.5) |
| --- | --- |
| $\lambda_{11}=E_{1}^{f}\left[ -E^{m}\left( 1-V_{f,t} \right)+K_{23}^{f}\left( 4v^{m}V_{f,t}-2V_{f,t}-2 \right)\left( 1+v^{m} \right) \right]+4E^{m}K_{23}^{f}v_{12}^{f}$  $(v^{m}-v_{12}^{f})(1-V_{f,t})$  $\lambda_{12}=4E^{m}K_{23}^{f}(v^{m}-v_{12}^{f})(1-V_{f,t})$  $\lambda_{13}=2\left( 1+v^{m} \right)\left[ \left( 2v_{12}^{f}-1 \right)V_{f,t}-1 \right]K_{23}^{f}E^{m}-E^{m^{2}}(1-V_{f,t})$  $\lambda_{21}=\left( 1-V_{f,t} \right)[\left( 2v^{m^{2}}E_{1}^{f}+4E^{m}v^{m}v_{12}^{f}+2E_{1}^{f}v^{m}-4E^{m}v_{12}^{f} \right)K_{23}^{f}+E_{1}^{f}E^{m}v^{m}]$  $\lambda_{22}=4[\left( v_{12}^{f}v^{m}-1 \right)\left( 1-V_{f,t} \right)E^{m}+E_{1}^{f}V_{f,t}\left( v^{m^{2}}-1 \right)]K_{23}^{f}$  $\lambda_{23}=-E^{m^{2}}\left( 1-V_{f,t} \right)-\{\left[ \left( 4v_{12}^{f^{2}}-4v_{12}^{f}-2 \right)K_{23}^{f}+E_{1}^{f} \right]V_{f,t}+2K_{23}^{f}\}(1+v^{m})E^{m}-2V_{f,t}E_{1}^{f}K_{23}^{f}{(1+v^{m})}^{2}$ |  |

**Appendix B. Tensor Transformation in Multiscale Modeling Framework**

A periodic sinusoid function (*f(x’)*) is employed to approximate the path of undulating fiber tows as,

| $z^{'}=f(x')$ | (B.1) |
| --- | --- |

From trigonometry, it is evident that

| $\tan\beta=\frac{df(x')}{dx'}$ | (B.2) |
| --- | --- |
| $\hat{m}=\cos\beta=\frac{1}{1+{tan}^{2}\beta}$ |  |
| $\hat{n}=\sin\beta=\frac{tan\beta}{1+{tan}^{2}\beta}$ |  |

And the transformation matrix for the thermal conductivity tensor from the local coordinate to the ply coordinate can be expressed as,

| ${\hat{\boldsymbol{T}}}_{1}=\left[ \begin{matrix} \hat{m} & 0 & \hat{n} \\ 0 & 1 & 0 \\ -\hat{n} & 0 & \hat{m} \end{matrix} \right]$ |
| --- |

Therefore, according to the microstructure parameters of the fiber tows (amplitude = 0.40mm, wavelength = 32.50mm ^[3]^), a sinusoid function is employed to approximate the undulating path of fiber tows, and the derivative of the function (tan*β*) with respect to *x* is included in the transformation tensor, which is used in the tensor transformation from the local coordinate into the ply coordinate (Eq. (9)). Meanwhile, based on fiber tow orientation *θ*, define

| $\hat{M}=\cos\theta=\frac{1}{1+{tan}^{2}\theta}$ | (B.3) |
| --- | --- |
| $\hat{N}=\sin\theta=\frac{tan\theta}{1+{tan}^{2}\theta}$ |  |

Then the transformation tensor for thermal conductivity tensor from the ply into the global coordinate ${\hat{\boldsymbol{T}}}_{2}$ can be expressed as,

| ${\hat{\boldsymbol{T}}}_{2}=\left[ \begin{matrix} \hat{M} & -\hat{N} & 0 \\ \hat{N} & \hat{M} & 0 \\ 0 & 0 & 1 \end{matrix} \right]$ |
| --- |

Similarly, for stiffness tensor, the transformation tensor from the local into the ply coordinate can expressed as,

$${\hat{\boldsymbol{T}}}_{1,m}=\left[ \begin{matrix} \begin{matrix} \hat{m}^{2} & 0 & \hat{n}^{2} \\ 0 & 1 & 0 \\ \hat{n}^{2} & 0 & \hat{m}^{2} \end{matrix} & \begin{matrix} 0 & 2\hat{m}\hat{n} & 0 \\ 0 & 0 & 0 \\ 0 & -2\hat{m}\hat{n} & 0 \end{matrix} \\ \begin{matrix} 0 & 0 & 0 \\ -2\hat{m}\hat{n} & 0 & -2\hat{m}\hat{n} \\ 0 & 0 & 0 \end{matrix} & \begin{matrix} \hat{m} & 0 & -\hat{n} \\ 0 & \hat{m}^{2}-\hat{n}^{2} & 0 \\ \hat{n} & 0 & \hat{m} \end{matrix} \end{matrix} \right]$$

and an ***R*** tensor is utilized to relate engineering strains to torsional strains as,

$$\boldsymbol{R}=\left[ \begin{matrix} \begin{matrix} 1 & 0 & 0 \\ 0 & 1 & 0 \\ 0 & 0 & 1 \end{matrix} & \begin{matrix} 0 & 0 & 0 \\ 0 & 0 & 0 \\ 0 & 0 & 0 \end{matrix} \\ \begin{matrix} 0 & 0 & 0 \\ 0 & 0 & 0 \\ 0 & 0 & 0 \end{matrix} & \begin{matrix} 2 & 0 & 0 \\ 0 & 2 & 0 \\ 0 & 0 & 2 \end{matrix} \end{matrix} \right]$$

and ${\hat{\boldsymbol{T}}}_{2,m}$ is the transformation tensor which can be applied to transform the stiffness tensor from the ply coordinate into the global coordinate as,

$${\hat{\boldsymbol{T}}}_{2,m}=\left[ \begin{matrix} \begin{matrix} \hat{M}^{2} & \hat{N}^{2} & 0 \\ \hat{N}^{2} & \hat{M}^{2} & 0 \\ 0 & 0 & 1 \end{matrix} & \begin{matrix} 0 & 0 & 2\hat{M}\hat{N} \\ 0 & 0 & -2\hat{M}\hat{N} \\ 0 & 0 & 0 \end{matrix} \\ \begin{matrix} 0 & 0 & 0 \\ 0 & 0 & 0 \\ -\hat{M}\hat{N} & \hat{M}\hat{N} & 0 \end{matrix} & \begin{matrix} \hat{M} & -\hat{N} & 0 \\ \hat{N} & \hat{M} & 0 \\ 0 & 0 & \hat{M}^{2}-\hat{N}^{2} \end{matrix} \end{matrix} \right]$$

**Reference**

1. Chen, W. & Zhang, D. A micromechanics-based processing model for predicting residual stress in fiber-reinforced polymer matrix composites. *Composite Structures* **204**, 153-166 (2018).
2. Zhang, D. & Anthony, M. Waas. A micromechanics based multiscale model for nonlinear composites. *Acta Mechanica* **225**, 1391-1417 (2014).
3. Kier, Z. T. et al. Estimating mechanical properties of 2D triaxially braided textile composites based on microstructure properties. *Composites Part B: Engineering* **68**, 288-299 (2015).
